# Characterization of Oil-in-Water Emulsions Prepared with Triblock Copolymer Poloxamer 407 and Low-Molecular-Mass Surfactant Mixtures as Carriers of Grape Pomace Waste Polyphenols

**DOI:** 10.3390/pharmaceutics16050578

**Published:** 2024-04-24

**Authors:** Veljko S. Krstonošić, Darija B. Sazdanić, Dejan M. Ćirin, Ivana R. Nikolić, Miroslav S. Hadnađev, Milica T. Atanacković Krstonošić

**Affiliations:** 1 Department of Pharmacy, Faculty of Medicine, University of Novi Sad, Hajduk Veljkova 3, 21000 Novi Sad, Serbia; 2 Faculty of Technology, University of Novi Sad, Bulevar Cara Lazara 1, 21000 Novi Sad, Serbia; 3 Institute of Food Technology, University of Novi Sad, Bulevar Cara Lazara 1, 21000 Novi Sad, Serbia

**Keywords:** O/W emulsion, rheology, stability, surfactant, extraction, grape pomace, polyphenols

## Abstract

Background: Natural antioxidants, such as grape pomace polyphenols, can be extracted by a surfactant-based green technology and incorporated into various emulsions. Therefore, this work aimed to investigate the physical stability and rheological characteristics of oil-in-water emulsions stabilized with poloxamer 407 (P407) and its mixtures with the low-molecular-mass surfactants Brij S20 (BS20) and Tween 60 (T60). Also, the influence of polyphenolic grape pomace extracts on the physical stability and rheological characteristics of the emulsions was examined. Methods: Grape pomace polyphenols were extracted by aqueous solutions of P407 and BS20/P407 and T60/P407 mixtures. Two different types of oil-in-water emulsions were examined: emulsions prepared with pure surfactants and emulsions prepared with surfactant-based polyphenol extracts of grape pomace. Both types contained 20% sunflower oil. Characterization of the emulsions comprised droplet size evaluation, rheology characteristics and creaming stability. Results: All the emulsions showed shear-thinning flow, while the rheological characteristics and creaming instability depended on the proportion of P407 in the emulsifier mixtures. Incorporation of grape pomace extracts had no effect on the investigated properties of the emulsions. Conclusion: The presence of extracted polyphenols in emulsifier mixtures had no significant effects on the emulsions’ physico-chemical characteristics and stability. Therefore, the investigated emulsions can be considered suitable carriers for polyphenol-rich extracts.

## 1. Introduction

Emulsions are thermodynamically unstable systems containing two phases (oil and water), with the diameters of the droplets being above the range of colloidal sizes (e.g., 100 nm–100 μm [1,2]). Unlike microemulsions, nanoemulsions and macroemulsions are thermodynamically unstable and show a tendency to break down over time through different processes (delamination of flocculation, coalescence, Ostwald ripening and phase inversion) and form separate liquid layers [1]. Emulsifiers (most often surfactants, but they can also be macromolecules or finely powdered solid components) have a role to play in ensuring the dispersion and stability of the system [3]. During the production of an emulsion, the emulsifier is adsorbed on the interface of the two phases, leading to a reduction of the surface tension, fragmentation of the droplets and enhancement of the system stability [4,5]. In addition, the emulsifier forms a protective layer on the dispersed droplets, which is important in the process of stabilization of emulsions [4]. It is known that the adequate stability of emulsions can only be achieved by using appropriate concentrations of surfactants; thus, most conventional creams and lotions usually contain between 2 and 7% (*w*/*w*) of these components [6]. 

Emulsions (conventional, microemulsions and nanoemulsions) are often used in the pharmaceutical and cosmetic industries as carriers of both hydrophilic and lipophilic substances [7]. The advantages of such systems include the increase in the water solubility and bioavailability of poorly soluble pharmaceutical active substances as well as promotion of the transdermal transport of hydrophilic compounds [7,8]. Incorporating active substances into emulsions enables combining both lipophilic and hydrophilic compounds within one formulation, protection of unstable substances as well as controlled release of active compounds [7,8,9,10]. 

The largest number of commercially available medicinal emulsions are intended for topical application. The type of emulsion is chosen based on the nature of the active substance and the indication for which the product is used. As topical carriers of active substances, emulsions can be an integral part of formulations such as lotions, creams, foams, emulgels, etc. Formulations based on emulsions are more suitable for application on the skin compared to other topical and transdermal dosage forms (such as solutions, ointments or patches). 

The surface layer of the skin (stratum corneum) consists of 10–20% lipids and 70–80% proteins, while water in normal conditions makes up 15–20%. Therefore, emulsions consisting of dispersed water and oil phase, are similar to the composition and conditions of the surface layer of the skin and, thus, have the greatest compatibility with the skin. Unlike ointments, topically applied emulsions do not prevent normal water loss and consequently do not cause discomfort. Emulsions are also easier to remove from the surface of the skin with water. Compared to solutions, the viscosity of emulsions can be more easily adjusted to prevent spillage of the formulation after application and to facilitate application to the desired, limited skin area [11]. 

Polyphenols are natural antioxidants, which can be incorporated into various cosmetic and pharmaceutical emulsions [9,12,13,14,15,16]. Topical application of polyphenolic antioxidants supports the endogenous antioxidant system of the skin and prevents the damage that can be caused by UV radiation [9,16]. One of the challenges when formulating a product containing polyphenols is the different polarity of these compounds, whereby some representatives have low solubility in water (e.g., quercetin, resveratrol, etc.) [16]. Therefore, emulsions are considered as suitable carriers for extracts rich in polyphenols. So far, it has been shown that polyphenolic compounds and their extracts can be effectively incorporated into O/W conventional emulsions, as well as micro- and nanoemulsions, using different homogenization techniques [9,17,18,19], while maintaining the chemical stability and antioxidant capacity of these compounds [20].

We have previously investigated the use of aqueous solutions of different types of non-ionic surfactants, as well as binary mixtures of surfactants, as mediums for the solid–liquid extraction of polyphenols from red grape pomace [21,22]. Polyoxyethylene (20) stearyl ether (Brij S20, BS20), polyoxyethylene (20) sorbitan monostearate (Tween 60, T60) and triblock copolymer poloxamer 407 (P407) have emerged as the most efficient representatives of each investigated surfactant group (i.e., Brij, Tween and poloxamer groups) [21], reaching the highest polyphenols content in the extracts. Binary mixtures of triblock copolymer P407 and the low-molecular-mass surfactants have also been proven to be efficient extraction mediums [22]. Therefore, the next phase was to prepare O/W emulsions with the aforementioned surfactants and their binary mixtures, which would serve as carriers of extracted polyphenols. In this way, surfactant-rich grape pomace polyphenolic extracts will be simultaneously used as emulsifiers (surfactants) and active substances (polyphenols). Also, these extract mixtures will be used without purification, which will reduce the energy and time consumption. 

Additionally, it is important to investigate the influence of polyphenols on the properties of emulsions stabilized with P407 and BS20 or T60. Namely, it is known that surfactants, i.e., emulsifiers, can interact with various compounds, even with the ones that are not surface-active [23,24,25]. In the same way, it can be expected that in the surfactant–polyphenols mixtures there is an interaction of polyphenols with individual molecules of the surfactants and/or with their micelles. These interactions, which can occur in the bulk of aqueous solutions and/or at the interfaces of two phases, can either decrease or increase the surface activity of the tensides [23,24,25]. As a result, the emulsifying properties of surfactants can be altered as well as the dispersion, stability and rheological properties of the emulsions [23]. 

Bearing in mind what has previously been stated, the aim of this work was to prepare emulsions stabilized with surfactant-rich polyphenolic grape pomace extracts (obtained with following surfactants: P407; binary mixtures BS20/P407 and T60/P407) and to investigate their dispersion and rheological characteristics as well as their physical stability. Also, the influence of the incorporated polyphenolic extracts on the emulsion characteristics was investigated and compared with the blank emulsions prepared without polyphenols. 

## 2. Materials and Methods

### 2.1. Materials

Sunflower oil was purchased from Bimal Sunce (Sombor, Serbia). Poloxamer 407 (Kolliphor^®^ P407) was donated by BASF Lampertheim GmbH (Lampertheim, Germany), polyoxyethylene (20) stearyl ether (Brij^®^ S20) was obtained from Sigma-Aldrich (Steinheim, Germany), while polyoxyethylene (20) sorbitan monostearate (Tween^®^ 60) was purchased from Fluka Chemie GmbH (Buchs, Switzerland). Double-distilled water was used for preparation of the solutions and emulsions. Sodium azide was purchased from Sigma-Aldrich (Steinheim, Germany).

### 2.2. Apparatus and Instruments

The following apparatus were used in the extraction and preparation of the emulsions: Hei-Standard MR 3001 magnetic stirrer (Heidolph Instruments, Schwabach, Germany), centrifuge (Centrifuge 2-5, Sigma, Osterode am Harz, Germany), InoLab pH meter (WTW, Weilheim, Germany), T25 digital Ultra-Turrax homogenizer, equipped with an S 25 N-18 G dispersive element (IKA, Staufen, Germany) and LHDM502 Universal Ultrasonic Generator Homogenizer (Colo, Novo Mesto, Slovenia). Measurements of the emulsion droplet size were conducted using a Mastersizer 2000 (Malvern Panalytical Ltd., Malvern, UK). The microphotographs were taken using an optical stereo microscope ZEISS STEMI 508 with a AXIOCM ERc 5s camera (Carl Zeiss Microscopy GmbH, Jena, Germany), while the obtained images were processed using ZenBlue Software 3.4 (White Plains, NY, USA) The determination of the rheological properties was performed using HAAKE MARS (Thermo Scientific, Karlsruhe, Germany). 

### 2.3. Grape Pomace Sample

Red grape pomace (Cabernet Franc variety) was obtained after a 15-day maceration by the “controlled wild fermentation” technique in a local winery. Afterwards, it was dehydrated by lyophilization, ground, and stored in a refrigerator at −20 °C until need for the extraction process.

### 2.4. Extraction Procedure

The sample was accurately weighed and the appropriate solvent systems were added to provide a solvent-to-material ratio of 100:1. The extraction was performed for 45 min at room temperature with constant stirring (300 rpm). The extracts were centrifuged, filtered through a membrane filter (0.45 μm) and collected for the preparation of the emulsions. Aqueous solutions of the non-ionic surfactants P407 and surfactant mixtures of the surfactants BS20/P407 and T60/P407 in concentrations above the critical micelle concentration (3% and 5% *w*/*v*) were used as solvent systems. The total phenolic content of the extracts has been investigated in previous research and was in the range from 28.45 ± 0.11 to 54.49 ± 0.12 mg GAE/g (mg of gallic acid equivalents per gram of grape pomace dry weight), depending on the applied surfactant or mixture as well as the surfactant concentration. The phenolic compounds in the extracts were characterized by high-performance liquid chromatography and phenolic acids (INCI names: gallic acid and syringic acid), flavan-3-ol (catechin), flavonols (INCI names: rutin, quercetin and kaempferol), flavanon (INCI name: naringenin) and stilbene (INCI name: resveratrol) were determined, while flavonol quercetin and flavn-3-ol catechin were the dominant. Also, the investigated aqueous solutions of surfactants and surfactant mixtures provided the extracts with significant radical scavenging activity [21,22].

### 2.5. Preparation of Emulsions

All the emulsions were prepared as O/W emulsions, containing 20% (*w*/*w*) sunflower oil, while the total mass of the emulsions was 100 g. The following emulsifiers were used: pure polymeric surfactant P407, as well as mixtures of the surfactants BS20/P407 and T60/P407, where the individual surfactants were mixed in mass ratios of 9:1, 1:1 and 1:9. The emulsion continuous phases were formed by adding surfactants in concentrations of 3% or 5% (*w*/*w*, calculated based on the mass of emulsion). In order to avoid microbiological contamination, 0.02% (*w*/*w*, calculated based on the mass of emulsion) of sodium azide was added to the continuous phase prior to the addition of the oil phase and homogenization. Primary homogenization was performed using a homogenizer Ultra-Turrax T25 digital, equipped with an S 25 N-18 G dispersing element, at room temperature at 10,000 rpm. The total primary homogenization time was 10 min. Secondary homogenization was carried out using an ultrasonic homogenizer LHDM502 with the frequency set to 20 kHz, while the total homogenization time was 5 min. 

Two types of emulsions have been prepared: control emulsions and test emulsions (Table 1). For the preparation of the control emulsions, the continuous phase was prepared by dissolving the appropriate surfactant/surfactant mixture in water, without added extract. In the production of the test emulsions, the continuous phase consisted of mixtures of grape pomace extracts and surfactants/surfactant mixtures. The appropriate mass of the same surfactants previously used for the extraction was added to the extracts in order to reach the desired concentrations of surfactants in the emulsions, as calculated based on the mass of the emulsion (3% or 5%, *w*/*w*).

### 2.6. Particle Size and Particle Size Distribution

The droplet size distribution was determined by the laser scattering method, using a Mastersizer 2000. The measurements were performed immediately after preparation of the emulsions, as well as after 14 days, at room temperature. Additionally, determination has been performed after centrifugation of the freshly prepared emulsion at 3000 rpm for 30 min. Each measurement was performed in triplicate and the average value was taken as a result. The results were analyzed using Malvern software 6.01 (Malvern Panalytical Ltd., Malvern, UK) and the droplet size distributions were reported using several parameters [26]:

- S—specific surface area,
- d_32_—surface-weighted mean (Sauter mean diameter),
- d_43_—volume-weighted mean,
- particle diameters—d (0.1), d (0.5) and d (0.9), which represent the mean mass diameters of the volume distribution and indicate that 10% of the sample is less than d (0.1), then 50% of the sample is less than d (0.5) and 90% of the sample is less than d (0.9),
- Span—the width of a droplet size distribution, calculated using the following Equation (1):

(1) $$\mathrm{Span}=\frac{d(0.9)-d(0.1)}{d(0.5)}$$

### 2.7. Microscopic Analysis

The microphotographs of the emulsions were taken using a stereo microscope ZEISS STEMI 508 with an AXIOCM ERc 5s camera in order to monitor the changes in the droplet size distribution. A drop of every emulsion was transferred to a glass slide covered by a cover slip and evaluated with an optical microscope coupled with a software for image analysis, ZenBlue Software 3.4. Three images were randomly taken for each emulsion. The images were observed at a magnification of 400×. The first recording time was immediately after the preparation of the emulsions and the second was after 14 days. At least 1000 droplets were counted per microphotograph of each control and test emulsion in order to determine the Sauter mean diameter (d_32_) using the following Equation (2):

(2) $$d_{32}=\frac{\sum n_{i}d_{i}^{3}}{\sum n_{i}d_{i}^{2}}$$

### 2.8. Rheological Properties

The rheological properties of the emulsions were determined using a HAAKE MARS rotational viscosimeter. All the measurements were performed with a cylinder DG41/Ti at a constant temperature of 25 ± 0.1 °C, 24 h after emulsion preparation. Before the measurements, the emulsions were left closed at room temperature, protected from evaporation of the aqueous phase. The rheological method included hysteresis loop tests. The emulsions were first exposed to an increasing shear rate from 0.001 to 100 s^−1^ during 120 s, followed by the constant shear rate of 100 s^−1^ for the next 60 s, and finally, the shear rate gradually decreased to 0 s^−1^ for the last 120 s. The flow curves were analyzed using a HAAKE RheoWin 4.91.0011 Data and Job Manager.

### 2.9. Creaming Stability

The creaming of the emulsions was monitored visually in 10 mL graduated cylinders. Upon preparation, the emulsions were immediately transferred to cylinders, sealed and left at room temperature. The extent of the creaming was characterized by the creaming index—CI (%) (Equation (3)):

(3) $$\mathrm{CI}\;(\%)=\frac{H_{s}}{H_{e}}\cdot 100$$

where H_s_ represents the volume of the transparent serum layer formed at the bottom of the cylinder and H_e_ is the total volume of the emulsion sample.

During the first 24 h after preparation, the creaming index was monitored at time intervals of 1 h. After that, the creaming index was monitored for the next 7 days by reading the values at 24 h intervals.

### 2.10. Determination of the pH Value of the Prepared Emulsions

The pH value of the control and test emulsions was determined at room temperature immediately after their preparation using an InoLab pH meter. 

### 2.11. Statistical Analysis

Statistical analysis of the data was performed and the significant differences at the significance level of 0.05 for several variables, based on three individual measurements, were determined by T-test and the ANOVA procedure (Tukey’s HSD post hoc test was used to determine the existence of statistical significance). The calculations were performed using the statistical software SPSS 22.0 (SPSS Inc., Chicago, IL, USA).

## 3. Results and Discussion

### 3.1. Dispersion Characteristics

At first, different parameters were analyzed in order to characterize the control emulsions stabilized with two different concentrations of P407 (3% and 5%, *w*/*w*). These two concentrations were also used for the polyphenol extraction from grape pomace. The parameters d_32_ and d_43_ as well as d (0.1), d (0.5) and d (0.9) were statistically significantly higher in the control emulsions stabilized with 3% (*w*/*w*) compared to 5% (*w*/*w*) P407. These results are in agreement with earlier studies, which found that using higher concentrations of different types of emulsifiers (e.g., different natural, low-molecular and polymeric emulsifiers) in O/W emulsions generates droplets with smaller diameters [27,28]. Zhang et al. (2017) analyzed the droplet sizes of O/W emulsions stabilized by three different concentrations of P407 (3%, 5% and 8%, *w*/*v*). At the highest concentration, the smallest droplets were obtained (130.30 ± 1.10 nm) [27]. The effect of the emulsifier concentration on the dimensions of the droplets of the internal phase can be explained through two mechanisms: (a) at higher concentrations, the number of emulsifier molecules that are available and can be adsorbed on the droplet surface during the homogenization process is greater; and (b) if the emulsifier is present in a higher concentration, the process of adsorption of the emulsifier molecules on the surface of the oil droplets is accelerated, which prevents the coalescence process more effectively [27,28].

Based on these results and the previous results obtained regarding the efficiency of the extraction of polyphenols from grape pomace at different concentrations of surfactants [21], for further examinations of the emulsifying abilities of P407 as well as P407/low-molecular-weight surfactants mixtures, the total mass concentration of the surfactant or surfactants mixtures of 5% (*w*/*w*) was chosen. The results of the particle size distribution as well as the d_32_ values obtained from the microphotographs are presented in Table 2 and Table 3. The results obtained from the droplet size distributions indicate that among the control emulsions stabilized with P407 and the BS20/P407 mixtures, the emulsion containing pure P407 had the lowest d_32_ value, while the statistically significantly lowest d_43_ value was recorded for the emulsion containing the BS20/P407 mixture (9:1). The emulsion stabilized with the mixture BS20/P407 (1:1) had the significantly highest d_32_ and d_43_ values (Table 2). On the other hand, in the second group of control emulsions, which contained P407 and T60/P407 mixtures as emulsifiers, the lowest d_32_ and d_43_ values were determined in the mixture T60/P407 (9:1), while higher values were observed in the emulsions with pure P407 (statistically significant differences are noticed regarding parameter d_43_) (Table 3). By comparing the d_32_ and d_43_ of the emulsions stabilized with BS20/P407 and T60/P407 mixtures, smaller droplet diameters are observed in the case of the T60/P407 surfactant mixtures. In the test emulsions (where the continuous phase consisted of grape pomace extracts), the d_32_ did not increase significantly compared to the corresponding control emulsions. An interesting phenomenon was observed in all the test emulsions stabilized with BS20/P407 mixtures, where a statistically significant decrease in the d_32_ was recorded compared to the corresponding control emulsions.

Based on the Span values obtained by measuring the droplet size distribution, it can be observed that the control emulsions stabilized with pure P407 and BS20/P407 (9:1) mixture have droplets of a more uniform size compared to the other emulsions stabilized with other mixtures of P407 and Brij S20. Also, the widest distribution is present in the BS20/P407 (9:1) (Table 2). On the other hand, in the case of the control emulsions stabilized with pure P407 or T60/P407 mixtures, the narrowest distribution is observed in the T60/P407 (9:1) emulsion. The emulsion with the broadest particle size distribution in this group was produced with pure P407 (Table 3). The Span value decreased in most of the test emulsions (emulsions with polyphenols extracts) compared to the corresponding control emulsions.

Figure S1 (in the Supplementary Materials) shows the particle size distributions of the control emulsions stabilized with pure P407 and BS20/P407 and T60/P407 mixtures. In general, bimodal distributions with one pronounced peak and another very small peak corresponding to droplet dimensions of about 10 µm are observed. A bimodal droplet size distribution was also observed in the emulsions stabilized by P407 in combination with nonionic (tetraethyleneglycol monododecyl ether), anionic (sodium dodecyl sulfate) and cationic (cetyltrimethylammonium bromide) co-surfactants [29].

No significant changes in the shape of the droplet size distribution curve or the peak numbers were observed in the test emulsions (with grape pomace extract as a continuous phase) compared to the corresponding control emulsions (selected results are presented in Figure S2 in the Supplementary Materials). It can also be noticed that the test emulsions generally have a smaller number of larger particles than the control emulsions, which is in agreement with the results presented in Table 2 and Table 3 (d (0.9) and Span values).

After 14 days at room temperature, the parameters d_32_ and d_43_ of the control emulsions and test emulsions did not change significantly. Also, after 14 days, no shift in the peak occurred, nor did additional peaks appear on the particle size distribution curve (selected results are presented in the Supplementary Materials in Figure S3). Therefore, it can be concluded that coalescence did not occur in the control emulsions or in the corresponding test emulsions during two weeks. Similarly, Kabong et al. (2020) examined the stability of emulsions over a period of 48 days at a temperature of 60 °C and observed that emulsions stabilized with 3.5 mM P407 and the nonionic co-surfactant tetraethylene glycol monododecyl ether in concentrations greater than 5 mM did not show changes in the particle size distribution. The same authors, on the other hand, observed that in the case of emulsions stabilized with P407 and anionic sodium dodecyl sulfate or cationic cetyltrimethylammonium bromide as co-surfactants, two or more peaks appeared over time, which corresponded to coalescence [29]. Additionally, analysis has been performed after centrifugation of the freshly prepared control and test emulsions and no statistically significant increase in the droplet size distribution parameters (d_32_ and d_43_) was observed. Namely, parameter d_43_ is sensitive to the formation of large droplets and, therefore, is often used to monitor the droplet size changes [30]. These results indicate that the applied emulsifiers have the ability to adsorb at the oil–water interface, creating a protective layer that stabilizes emulsions for a longer period of time without the occurrence of coalescence—a phenomenon that directly leads to phase separation. Moreover, the presence of grape pomace phenolic compounds does not affect the ability of surfactants to form an effective layer that stabilizes emulsions. Consequently, the mixture of surfactants and polyphenols obtained after the extraction, i.e., the whole extract, can be directly used in the preparation of stable emulsions.

**Figure 1:**
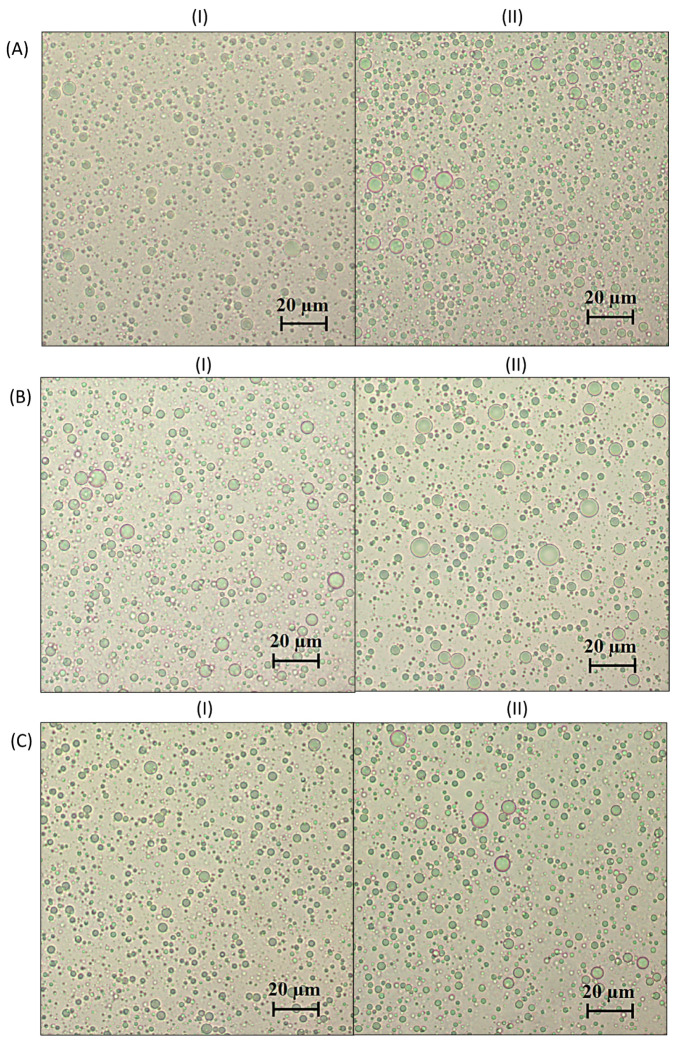
Microphotographs of control (**I**) and test emulsions (**II**) stabilized with: (**A**) P407, (**B**) BS20/P407 (1:9) and (**C**) T60/P407 (9:1).

Microphotographs of selected control and corresponding test emulsions are presented in Figure 1, while the d_32_ values obtained from microphotographs are presented in Table 2 and Table 3. 

The values of d_32_ obtained from the microphotographs are slightly higher than those obtained from the droplet size distributions (Table 2 and Table 3). The observed differences in the results can be explained by the limitations of detecting and measuring droplets smaller than approximately 1 μm by means of microphotography at the used magnification. However, a similar pattern as seen for the particle size distribution parameters obtained by the light-scattering method was observed (e.g., smaller droplet diameters are observed in the case of the control emulsions stabilized with T60/P407 compared to BS20/P407). Also, in the microphotographs taken 14 days after preparation of the emulsions, flocculation was not observed in any of the analyzed systems. On the other hand, the polydispersity was observed in all the tested surfactant systems.

### 3.2. Rheological Characteristics

**Figure 2:**
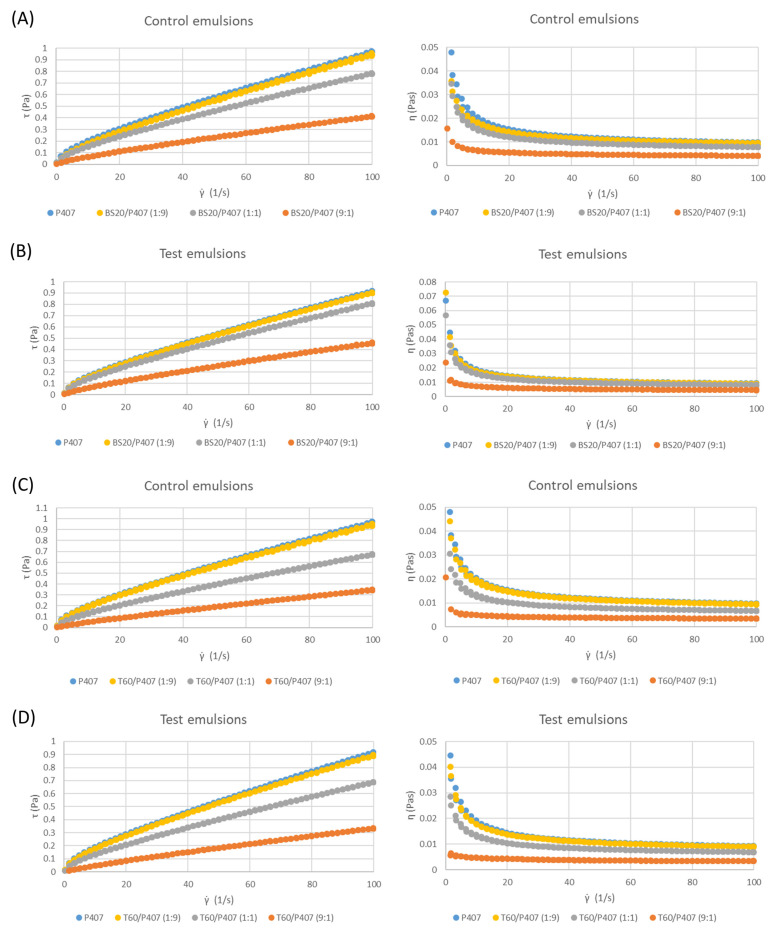
Shear stress and apparent viscosity versus shear rate curves for the 5% (*w*/*w*) emulsions stabilized with P407 and mixtures BS20/P407 (**A**) control and (**B**) test emulsions and with P407 and mixtures T60/P407 (**C**) control emulsions and (**D**) test emulsions.

All the tested emulsions (control and test samples) showed shear-thinning flow behavior with the overlapping of the downward and upward curves (Figure 2). Also, none of the tested emulsions showed a linear viscoelastic region, which indicates that the systems did not show yield stress, which is obvious from flow curves at logarithmic-scale figures. The rheological characteristics of emulsions are usually described using mathematical models. There are several models, such as Ostwald–de Waele (power law), Casson, Herschel–Bulkley and other models, that can be used to describe the characteristics of the emulsions’ flow curves [31]. In this paper, the Herschel–Bulkley model, which provides information on the existence of yield stress, and the power-law model, which refers to the emulsions that do not show yield stress, were chosen and used to fit the flow curves of the investigated emulsions. The correlation coefficients after fitting the curves had higher values for the power-law model than in the case of the Herschel–Bulkley model [32], which is another confirmation that the emulsions do not possess yield stress. Therefore, the power-law model was suitable for the determination of the flow parameters of the emulsions (Equation (4)):

(4) $$\tau =K{\dot{\gamma }}^{n}$$

where τ (Pa) is the shear stress, $\dot{\gamma }\;(s^{-1})$ is the shear rate, K (Pa $s^{n})$ is a consistency index and n (no dimensional) is the flow behavior index.

The fitting results for the emulsions obtained by Equation (4) for the emulsion flow curves (Figure 2) are presented in Table 4 and Table 5. All the results were statistically analyzed by the ANOVA test. The results from Table 4 and Table 5 confirm the shear-thinning character of all the control and test emulsions, regardless of the used emulsifier, based on the value of the flow behavior index *n* (in all cases, the values of this index were lower than 1). Moreover, it can be noticed that there was an increase in non-Newtonian behavior (the decrease in the flow behavior index *n*) with an increase in the proportion of P407 in all the tested emulsions, which is also visible in Figure 2. Also, the values of the consistency index *K* increase with the increase in the proportion of P407, indicating an increase in the apparent viscosity of the system. Namely, since P407 is a macromolecular emulsifier, in addition to its emulsifying properties due to its amphiphilic nature, it increases the viscosity of the continuous phase of O/W emulsions as well. The influence of P407 on the aqueous phase of the emulsions is greater compared to classic low-molecular emulsifiers (BS20 and T60). By replacing part of the content of P407 with emulsifiers of low molecular weight, whose influence on viscosity is negligible, the viscosity of the continuous phase was lowered, and thus the viscosity of the entire system became lower. It was previously established that the flow of solutions of hydroxypropylmethylcellulose, methylcellulose, gelatin, P407 and poloxamer 188 exhibits a non-Newtonian character, with solutions of macromolecules with long and twisted chains having a higher viscosity. Zhang et al. (2017) also found that the viscosity of the continuous phase (macromolecular emulsifier solution) has the most pronounced influence on the physical stability of emulsions, while the droplet size of the internal phase and surface tension have a less pronounced influence [27]. Dokić et al. (2008) examined the rheological characteristics of emulsions stabilized with macromolecular OSA starch, Tween 80 and their mixture and determined that the emulsion stabilized with macromolecular emulsifier had the highest consistency index *K*, while the emulsion stabilized with pure Tween 80 had the lowest *K* values, which indicates the lowest viscosity [33]. Also, with an increase in the proportion of sodium lauryl sulfate in the mixtures with OSA starch as an emulsifier, a decrease in the consistency index, i.e., the apparent viscosity of the O/W emulsions, was observed [34].

**Figure 3:**
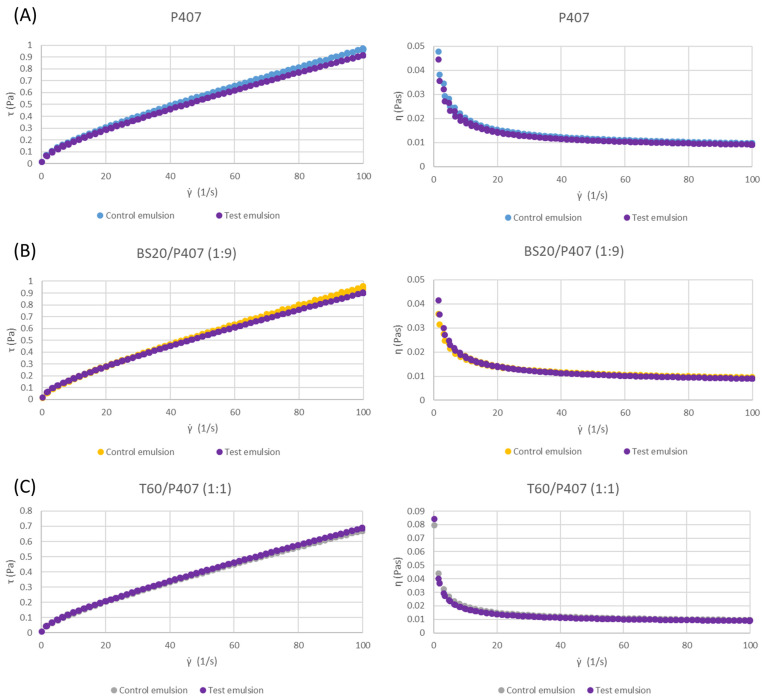
Shear stress and apparent viscosity versus shear rate curves for the 5% (*w*/*w*) control and corresponding test emulsions stabilized with: (**A**) P407, (**B**) BS20/P407 (1:9) and (**C**) T60/P407 (1:1).

The values of the consistency index K and flow behavior index *n* are not significantly different for the control emulsions and emulsions with pomace extract as the continuous phase (test samples), which were prepared using the same emulsion system, except in the case of the BS20/P407 (1:1) system (Table 4). Therefore, based on the values given in Table 4 and Table 5, as well as on the basis of the selected flow curves (Figure 3), it can be concluded that grape pomace extract rich in polyphenols does not significantly affect the viscosity and rheological characteristics of emulsions. This result is important because it indicates that mixtures of surfactants and polyphenol-rich extracts can be used to prepare emulsions that will serve as carriers of polyphenols as active agents.

### 3.3. Emulsion Creaming Stability

Creaming is one of the processes that lead to the physical instability of O/W emulsions, and it occurs under the influence of gravity force due to the difference in the phase densities [4]. The changes in the creaming index by direct observation of the emulsion separation in graduated cylinders were measured within 7 days.

The effect of the emulsifier concentration on the creaming index was examined in the control emulsions stabilized with P407. Emulsifier concentrations of 3% and 5% (*w*/*w*), which are in the range of concentrations that were examined in earlier studies [27,35], were examined. Figure S4 (Supplementary Materials) shows the significant creaming instability of the tested emulsions (both concentrations 3% and 5%, *w*/*w*), but the different stability patterns of the emulsions prepared with different emulsifier concentrations were already observed during the first hour of monitoring. Namely, a concentration of 5% (*w*/*w*) led to the greater stability of the emulsions and, therefore, was selected for further testing. It is expected that a higher concentration of macromolecular surfactant improves the creaming stability, but here the phenomenon is noticeable even in the first 24 h.

According to Stokes’ law, the velocity of moving a spherical particle through a Newtonian fluid, i.e., the speed of the droplets’ creaming, depends on the square of the particle diameter, the difference in the particle and fluid densities, as well as the fluid viscosity [36] (Equation (5)):

(5) $$w=\frac{d^{2}g∆\rho }{18\eta }$$

Therefore, larger droplets move faster compared to smaller droplets, i.e., they have a higher chance of floating. A reduction in the number of larger droplets in emulsions, as well as an increase in the viscosity of the continuous phase, slows down the movement of the droplets, leading to the higher stability of the emulsions (slower or delayed onset of creaming). Therefore, the greater instability of the emulsions with a lower emulsifier concentration (Figure S4, Supplementary Materials) is in agreement with the aforementioned significantly larger d_32_ and d_43_ values. Also, the greater stability of the emulsion with a higher concentration of P407 is in agreement with the influence of this macromolecule on increasing the viscosity of the continuous phase.

**Figure 4:**
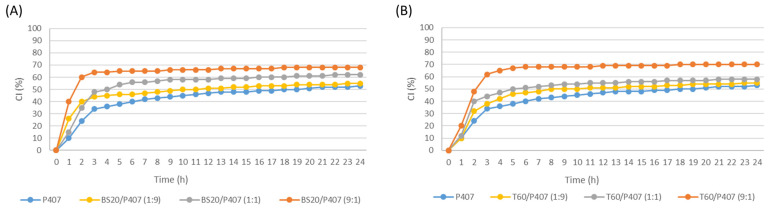
Creaming index values of the 5% (*w*/*w*) control emulsions stabilized with the following emulsifiers: (**A**) P407 and mixtures of P407 with Brij S20, (**B**) P407 and mixtures of P407 with Tween 60 (monitored for the first 24 h).

In order to examine the influence of the type of emulsifier on the stability of the emulsions, the creaming index of the control emulsions made with the use of the following emulsifier systems was monitored (concentration 5% (*w*/*w*)): P407, BS20/P407 (1:9), BS20/P407 (1: 1), BS20/P407 (9:1), T60/P407 (1:9), T60/P407 (1:1) and T60/P407 (9:1). Figure 4 shows the instability of all the tested control emulsions, which occurs during the first 24 h after the preparation of the emulsions. Although Stokes’ law shows that the droplet velocity (i.e., creaming) depends on the diameter [36], no correlation was observed between the d_32_ and d_43_ values (Table 2 and Table 3) and the creaming index of the tested emulsions with different proportions of individual emulsifiers. Therefore, it can be assumed that the main reason for the fast onset of creaming of the tested O/W emulsions is the insufficient viscosity of the continuous phase (Table 4 and Table 5). Since poloxamers are nonionic surfactants, it is considered that the stabilization of the emulsions is provided by a steric mechanism due to the existence of voluminous polar groups of molecules, which are adsorbed on the surface of oil droplets and strongly hydrated in the continuous phase, and they form layers that do not allow droplets to join and form aggregates [37]. In Figure 4, it can be observed that with the increase in the proportion of the macromolecular emulsifier P407 in the emulsion, its stability increases (i.e., lower values of the creaming index were recorded during the monitoring period of 24 h). This phenomenon can be explained by the increase in the viscosity of the continuous phase in the emulsions with an increase in the poloxamer content, since it is a macromolecule that significantly increases its viscosity when dissolved in water, which is confirmed by the values of the consistency index, as shown in Table 4 and Table 5. Moreover, by comparing the values of the creaming index for the control emulsions made using BS20/P407 and the corresponding T60/P407 mixtures, no significant effects of the emulsifier system on the stability of the emulsions are observed (Figure 4). Namely, the creaming of these emulsions could be slowed down and potentially stopped by adding hydrocolloids of large molecular masses, which increase the viscosity and modify the rheological characteristics of the continuous phase [38,39].

During a monitoring period of 7 days, all the control emulsions showed slight changes in the creaming index compared to the value recorded after the first 24 h (Figure S5, Supplementary Materials).

**Figure 5:**
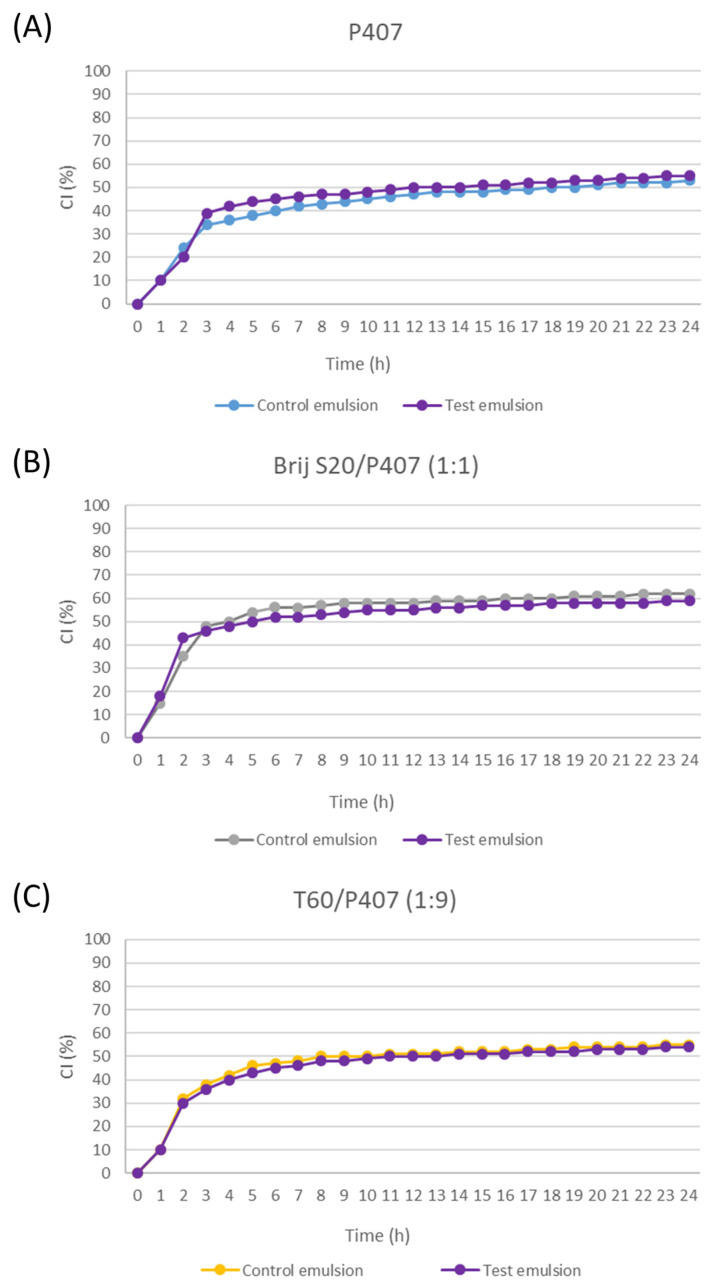
Creaming index values of the 5% (*w*/*w*) control and test emulsions stabilized with: (**A**) P407, (**B**) mixture BS20/P407 (1:1) and (**C**) mixture T60/P407 (1:9) (monitored for the first 24 h).

After incorporating grape pomace extracts into the continuous phase of the emulsions, it was observed that the extract had no effect on the changes in the creaming index during 24 h (Figure 5). Since there was no statistically significant increase in the d_32_ in the emulsions with pomace extract, no changes in the distribution of the particle sizes, nor in general a significant increase in the value of the consistency coefficient K, the absence of differences in the stability of the control and test emulsions is in line with expectations. The obtained results therefore indicate that the emulsions can be carriers of polyphenols as active substances, since the incorporation of grape pomace extracts into the emulsion models did not lead to changes in the dispersion and rheological characteristics or in the physical stability of the system. Also, it was previously established that the incorporation of water-ethanol apple extract into O/W emulsions in a total proportion of 6% (*w*/*w*) does not impair the physico-chemical stability of the emulsions (emulsions after exposure to centrifugation, pH value and emulsion viscosity were monitored) [40]. In addition, it was demonstrated that the incorporation of extracts rich in polyphenols can also increase the chemical stability of the extracts. Namely, incorporation of the water-ethanol extract in increasing concentrations (from 0.06% to 3% (*v*/*v*)) into O/W emulsions stabilized with Tween 20 (1%) was found to achieve greater inhibition of lipid peroxidation in the systems [41]. This is a good basis for further development of the formulation containing the prepared emulsion as a carrier of polyphenols. The first steps in the formulation development should be focused on increasing the creaming stability by adding thickeners, etc.

**Figure 6:**
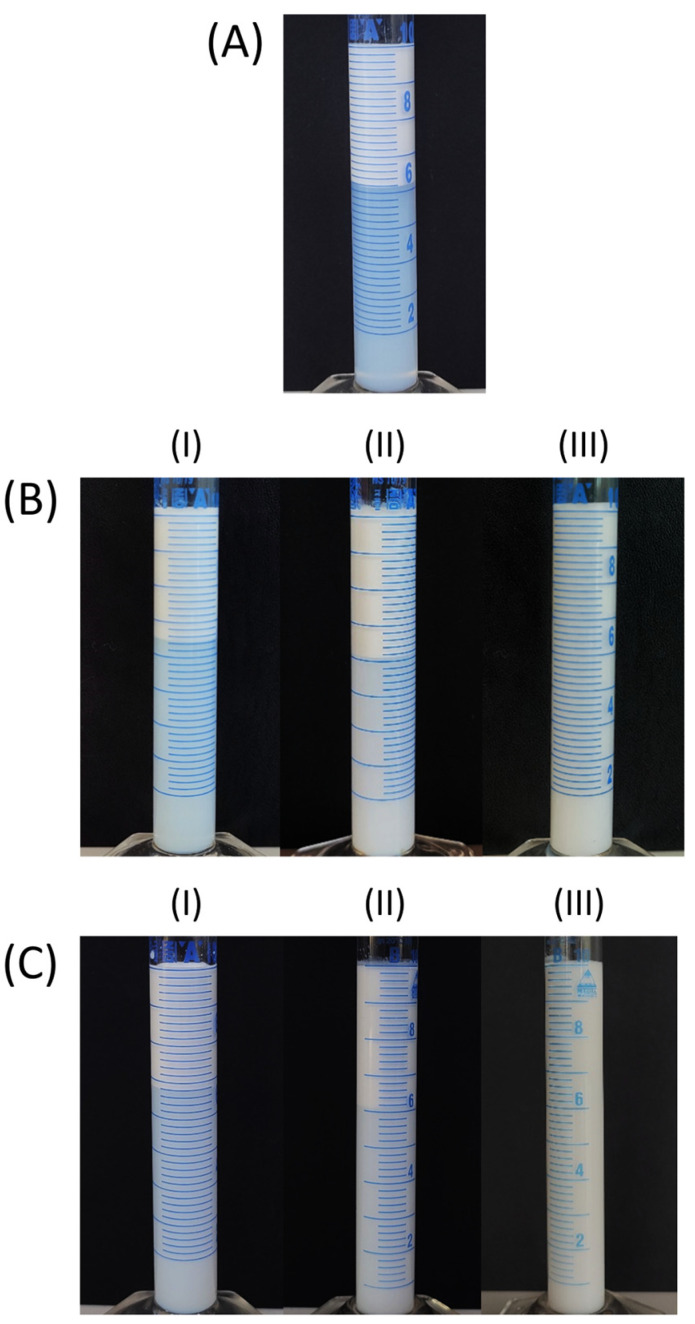
Creaming index of the 5% (*w*/*w*) control emulsions stabilized with: (**A**) P407; (**B**) mixtures of BS20/P407 (mass ratios (**I**) 1:9, (**II**) 1:1 and (**III**) 9:1); (**C**) mixtures of T60/P407 (mass ratios (**I**) 1:9, (**II**) 1:1 and (**III**) 9:1).

**Figure 7:**
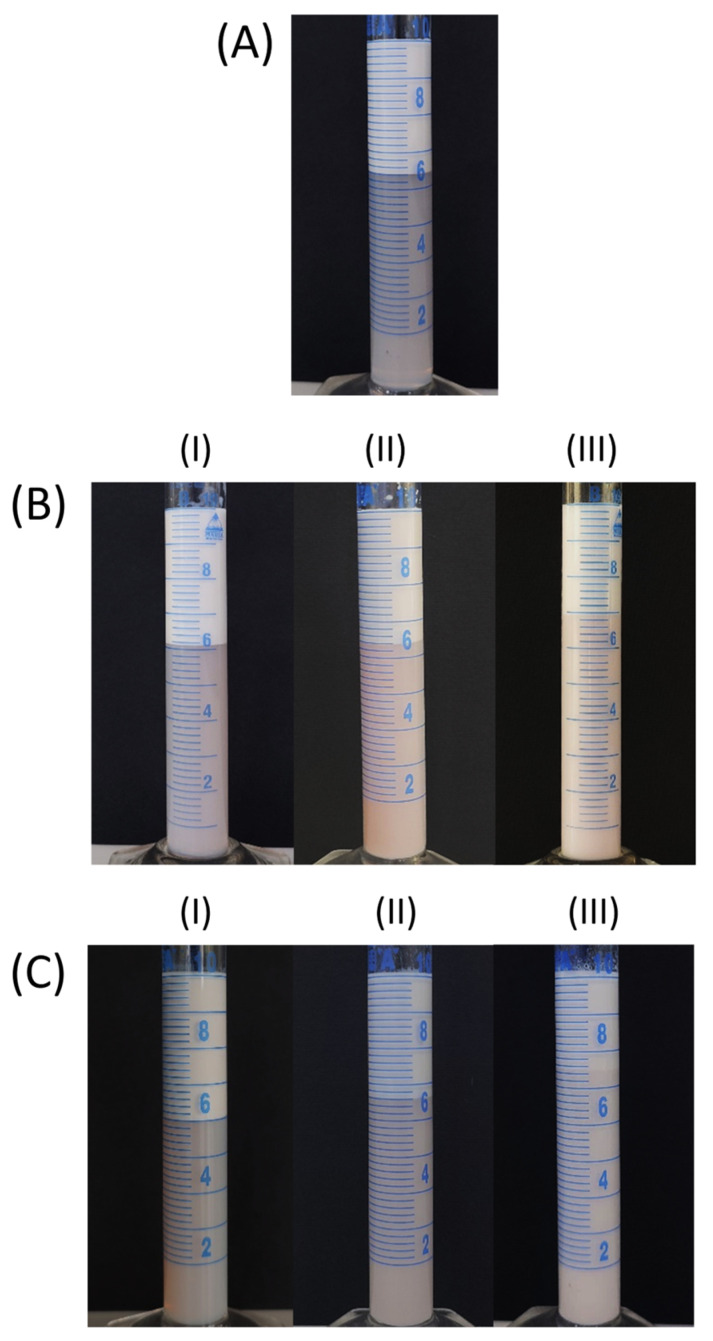
Creaming index of the 5% (*w*/*w*) test emulsions stabilized with: (**A**) P407; (**B**) mixtures of BS20/P407 (mass ratios (**I**) 1:9, (**II**) 1:1 and (**III**) 9:1); (**C**) mixtures of T60/P407 (mass ratios (**I**) 1:9, (**II**) 1:1 and (**III**) 9:1).

Figure 6 and Figure 7 present photographs of the control and test emulsions, respectively, after 24 h of storage. A clear boundary between the two layers can be observed in most cases. The upper layer is enriched with oil droplets and turbid, while the bottom layer is depleted of the oil droplets, and it is less turbid in all the emulsions except the control emulsions stabilized with BS20/P407 (9:1) and T60/P407 (9:1), in which the bottom layer was slightly turbid (Figure 6). In the case of the test emulsions (containing grape pomace extracts), the bottom layer is colored due to the presence of anthocyanins in the extracts and a clear boundary is observed (Figure 7).

### 3.4. Determination of the pH Value of the Emulsions

According to the literature data, the normal pH of the skin surface of most body parts is in the range of pH 4.1–5.8 (with the exception of physiological gaps, including axillae, groin, toe interdigits and anus, exhibiting pH values between 6.1 and 7.4) [42]. Moreover, there is a high level of agreement that topical products should be acidified and possess a pH in the range of 4 to 6 [43]. The pH value of the prepared control emulsions (prepared with distilled water as a continuous phase) varied between 6.10 and 6.40, while in the test emulsions with incorporated extracts, the pH ranged from 5.20 to 5.40. Therefore, the pH of the control and test emulsions can be considered appropriate for topical application. 

## 4. Conclusions

In this paper, the oil-in-water emulsions prepared using the macromolecular emulsifier P407 and mixtures of P407 with the low-molecular-weight surfactants BS20 and T60 were investigated. They showed adequate long-term physical stability regarding the occurrence of oil droplet coalescence. This indicates that the used surfactants are capable of adsorbing at the oil–water interface, create an effective molecular layer and ensure the stability of the dispersed droplets. Also, the presence of the polyphenols together with the surfactants did not affect the effectiveness of the applied surfactants in providing the stability for the emulsions to coalescence. Additionally, the creaming stability of the observed emulsions was not satisfactory, which is a consequence of the insufficient viscosity of the continuous phase in the O/W emulsions. Such instability, which does not lead to phase separation, nevertheless indicates the possibility of improving the formulation. All the emulsions showed shear-thinning flow behavior. This type of flow, which is common for solutions of macromolecules and emulsions, is especially suitable for topical formulations, as it enables the good spreadability of the preparation. Also, it was observed that the rheological characteristics and creaming instability of the emulsions depended on the proportion of macromolecular emulsifier in the emulsifying mixture. Namely, the emulsions with a higher content of P407 showed higher degrees of non-Newtonian flow behavior as well as lower values of creaming indexes. Incorporation of surfactant-rich grape pomace extracts into the continuous phase of the emulsions had no effect on the tested properties, such as the dispersion properties, physical stability and rheological characteristics. However, an interesting phenomenon was noticed: a statistically significant reduction in particle size was recorded for the BS20/P407 emulsions containing polyphenols compared to the corresponding control emulsions. Therefore, from the standpoint of stability and rheology, O/W emulsions can be considered suitable carriers for polyphenol-rich extracts.

## Figures and Tables

**Table 1:** Types of prepared and tested emulsions.

Emulsion Type	Continuous Phase	Surfactant Concentration (*w*/*w*, %)
Control emulsion	Water	3
		5
Test emulsion	Grape pomace extract	3
		5

**Table 2:** The droplet size distribution parameters of the control and corresponding test emulsions (emulsions with grape pomace extracts incorporated into the continuous phase) stabilized with pure P407 and BS20/P407 mixtures.

Surfactant System	Specific Surface Area S (m^2^/g)	Surface-Weighted Mean d_32_ (μm)	Volume-Weighted Mean d_43_ (μm)	d (0.1) (μm)	d (0.5) (μm)	d (0.9) (μm)	Span	Uniformity	d_32_ (μm) Determined Using Microphotography ^1^
	Control emulsions								
P407	3.717 ± 0.023 ^abd^	1.614 ± 0.011 ^abd^	3.702 ± 0.084 ^ab^*	0.788 ± 0.005 ^ab^	1.945 ± 0.012 ^abd^	8.973 ± 0.601 ^ad^*	4.212 ± 0.287 ^ab^*	1.293 ± 0.038 ^ac^*	2.25 ± 0.11
BS20/P407 (1:9)	3.583 ± 0.070 ^abd^*	1.677 ± 0.033 ^abd^*	3.557 ± 0.187 ^ab^*	0.808 ± 0.011 ^abd^*	2.047 ± 0.049 ^abd^*	8.793 ± 0.663 ^b^*	3.898 ± 0.238 ^ab^*	1.130 ± 0.065 ^b^*	2.28 ± 0.13
BS20/P407 (1:1)	3.277 ± 0.099 ^c^*	1.833 ± 0.054 ^c^*	4.240 ± 0.150 ^c^*	0.863 ± 0.019 ^cd^	2.279 ± 0.088 ^c^*	10.648 ± 0.485 ^c^*	4.295 ± 0.044 ^c^*	1.273 ± 0.011 ^ac^*	2.34 ± 0.14
BS20/P407 (9:1)	3.603 ± 0.075 ^abd^*	1.666 ± 0.033 ^abd^*	3.034 ± 0.147 ^d^*	0.836 ± 0.014 ^bcd^*	2.027 ± 0.039 ^abd^*	5.973 ± 0.334 ^ad^*	2.533 ± 0.113 ^d^*	0.877 ± 0.047 ^d^*	2.29 ± 0.09
	Test emulsions								
P407	3.950 ± 0.166 ^efgh^	1.520 ± 0.062 ^efgh^	3.010 ± 0.258 ^efg^*	0.752 ± 0.024 ^efgh^	1.843 ± 0.079 ^efgh^	6.341 ± 0.744 ^efg^*	3.024 ± 0.273 ^ef^*	1.019 ± 0.083 ^ef^*	2.23 ± 0.07
BS20/P407 (1:9)	3.960 ± 0.105 ^efgh^*	1.516 ± 0.039 ^efgh^*	2.769 ± 0.129 ^efg^*	0.756 ± 0.020 ^efgh^*	1.842 ± 0.053 ^efgh^*	5.721 ± 0.024 ^efg^*	2.697 ± 0.090 ^efg^*	0.890 ± 0.104 ^efg^*	2.24 ± 0.1
BS20/P407 (1:1)	3.803 ± 0.150 ^efgh^*	1.579 ± 0.062 ^efgh^*	2.682 ± 0.038 ^efgh^*	0.799 ± 0.038 ^efgh^	1.915 ± 0.066 ^efgh^*	5.418 ± 0.187 ^efg^*	2.412 ± 0.082 ^fg^*	0.779 ± 0.033 ^fgh^*	2.24 ± 0.11
BS20/P407 (9:1)	3.993 ± 0.100 ^efgh^*	1.503 ± 0.037 ^efgh^*	2.344 ± 0.074 ^gh^*	0.769 ± 0.022 ^efgh^*	1.803 ± 0.076 ^efgh^*	4.397 ± 0.134 ^h^*	1.980 ± 0.020 ^h^*	0.643 ± 0.014 ^gh^*	2.23 ± 0.12

^1^ Surface-weighted mean (d_32_ (μm)) was determined from microphotographs of control and corresponding test emulsions. ^a–d^ Mean ± standard deviation (*n* = 3) of control emulsions prepared with different emulgators, different letters in columns designate statistically significant differences (*p* < 0.05). ^e–h^ Mean ± standard deviation (*n* = 3) of test emulsions prepared with different emulgators, different letters in columns designate statistically significant differences (*p* < 0.05). * Mean ± standard deviation (*n* = 3) of control and corresponding test emulsions prepared with different emulgators that are significantly different (*p* < 0.05).

**Table 3:** The droplet size distribution parameters of the control and corresponding test emulsions (emulsions with grape pomace extracts incorporated into the continuous phase) stabilized with pure P407 and T60/P407 mixtures.

Surfactant System	Specific Surface Area S (m^2^/g)	Surface Weighted Mean d_32_ (μm)	Volume Weighted Mean d_43_ (μm)	d (0.1) (μm)	d (0.5) (μm)	d (0.9) (μm)	Span	Uniformity	d_32_ (μm) Determined Using Microphotography ^1^
	Control emulsions								
P407	3.717 ± 0.023 ^abcd^	1.614 ± 0.011 ^abc^	3.702 ± 0.084 ^a^*	0.788 ± 0.005 ^abcd^	1.945 ± 0.012 ^abc^	8.973 ± 0.601 ^a^*	4.212 ± 0.287 ^a^*	1.293 ± 0.038 ^a^*	2.25 ± 0.14
T60/P407 (1:9)	3.937 ± 0.176 ^abcd^	1.526 ± 0.066 ^abcd^	2.860 ± 0.196 ^bc^	0.760 ± 0.028 ^abcd^	1.843 ± 0.079 ^abcd^	6.310 ± 0.701 ^bc^	3.004 ± 0.242 ^b^*	0.932 ± 0.445 ^b^	2.23 ± 0.11
T60/P407 (1:1)	3.770 ± 0.065 ^abcd^	1.591 ± 0.029 ^abcd^	2.602 ± 0.147 ^bcd^	0.817 ± 0.012 ^abcd^	1.918 ± 0.052 ^abcd^	5.173 ± 0.365 ^bcd^	2.270 ± 0.137 ^cd^*	0.728 ± 0.062 ^cd^*	2.25 ± 0.12
T60/P407 (9:1)	3.990 ± 0.101 ^abcd^	1.504 ± 0.038 ^bcd^	2.244 ± 0.090 ^cd^*	0.801 ± 0.039 ^abcd^*	1.799 ± 0.024 ^bcd^	3.991 ± 0.188 ^cd^*	1.775 ± 0.143 ^cd^*	0.603 ± 0.074 ^cd^*	2.22 ± 0.12
	Test emulsions								
P407	3.950 ± 0.166 ^efgh^	1.520 ± 0.062 ^efgh^	3.010 ± 0.258 ^efg^*	0.752 ± 0.024 ^efgh^	1.843 ± 0.079 ^efgh^	6.341 ± 0.744 ^efg^*	3.024 ± 0.273 ^ef^*	1.019 ± 0.083 ^e^*	2.23 ± 0.1
T60/P407 (1:9)	3.933 ± 0.038 ^efgh^	1.525 ± 0.015 ^efgh^	2.755 ± 0.055 ^efgh^	0.766 ± 0.007 ^efgh^	1.848 ± 0.014 ^efgh^	5.511 ± 0.135 ^efgh^	2.567 ± 0.055 ^fg^*	0.871 ± 0.024 ^fg^	2.23 ± 0.08
T60/P407 (1:1)	3.797 ± 0.231 ^efgh^	1.584 ± 0.093 ^efgh^	2.851 ± 0.146 ^efgh^	0.782 ± 0.048 ^efgh^	1.804 ± 0.367 ^efgh^	5.930 ± 0.470 ^efg^	2.630 ± 0.079 ^fg^*	0.848 ± 0.019 ^fgh^*	2.24 ± 0.11
T60/P407 (9:1)	4.017 ± 0.030 ^efgh^	1.493 ± 0.011 ^efgh^	2.518 ± 0.134 ^fgh^*	0.756 ± 0.009 ^efgh^*	1.832 ± 0.008 ^efgh^	4.731 ± 0.072 ^fh^*	2.169 ± 0.029 ^h^*	0.754 ± 0.010 ^gh^*	2.20 ± 0.1

^1^ Surface-weighted mean (d_32_ (μm)) was determined from microphotographs of control and corresponding test emulsions. ^a–d^ Mean ± standard deviation (*n* = 3) of control emulsions prepared with different emulgators, different letters in columns designate statistically significant differences (*p* < 0.05). ^e–h^ Mean ± standard deviation (*n* = 3) of test emulsions prepared with different emulgators, different letters in columns designate statistically significant differences (*p* < 0.05). * Mean ± standard deviation (*n* = 3) of control and corresponding test emulsions prepared with different emulgators that are significantly different (*p* < 0.05).

**Table 4:** Consistency index K and flow behavior index *n* obtained by fitting the flow curves of the BS20/P407 control and test emulsions with Equation (4).

Surfactant System	K (Pa s^n^)	*n*
	Control emulsions	
P407	0.03256 ± 0.00198 ^a^	0.71847 ± 0.0087 ^ac^
BS20/P407 (1:9)	0.02954 ± 5.42·10^−4 b^	0.74470 ± 0.0038 ^bc^
BS20/P407 (1:1)	0.02633 ± 3.65·10^−4 c^*	0.73363 ± 0.0015 ^abc^*
BS20/P407 (9:1)	0.00965 ± 7.68·10^−4 d^	0.81877 ± 0.0076 ^d^
	Test emulsions	
P407	0.03541 ± 0.00144 ^e^	0.71503 ± 0.0067 ^efg^
BS20/P407 (1:9)	0.03137 ± 0.0025 ^fg^	0.72497 ± 0.0121 ^efg^
BS20/P407 (1:1)	0.02878 ± 5.06·10^−4 fg^*	0.72393 ± 0.0048 ^efg^*
BS20/P407 (9:1)	0.00978 ± 7.72·10^−4 h^	0.82220 ± 0.0072 ^h^

^a–d^ Mean ± standard deviation (*n* = 3) of control emulsions prepared with different emulgators, different letters in columns designate statistically significant differences (*p* < 0.05). ^e–h^ Mean ± standard deviation (*n* = 3) of test emulsions prepared with different emulgators, different letters in columns designate statistically significant differences (*p* < 0.05). * Mean ± standard deviation (*n* = 3) of control and corresponding test emulsions prepared with different emulgators that are significantly different (*p* < 0.05).

**Table 5:** Consistency index K and flow behavior index *n* obtained by fitting the flow curves of the T60/P407 control and test emulsions with Equation (4).

Surfactant System	K (Pa s^n^)	*n*
	Control emulsions	
P407	0.03256 ± 0.00198 ^ab^	0.71847 ± 0.00866 ^abc^
T60/P407 (1:9)	0.02801 ± 0.00644 ^abc^	0.72711 ± 0.04786 ^abc^
T60/P407 (1:1)	0.02296 ± 3.76·10^−4 bc^	0.72803 ± 0.00404 ^abc^
T60/P407 (9:1)	0.00617 ± 5.71·10^−4 d^	0.86697 ± 0.01023 ^d^
	Test emulsions	
P407	0.03541 ± 0.00144 ^ef^	0.71503 ± 0.00675 ^efg^
T60/P407 (1:9)	0.03297 ± 0.00191 ^ef^	0.71907 ± 0.00841 ^efg^
T60/P407 (1:1)	0.02274 ± 2.41·10^−4 g^	0.73827 ± 0.00352 ^efg^
T60/P407 (9:1)	0.00697 ± 6.62·10^−4 h^	0.83987 ± 0.02105 ^h^

^a–d^ Mean ± standard deviation (*n* = 3) of control emulsions prepared with different emulgators, different letters in columns designate statistically significant differences (*p* < 0.05). ^e–h^ Mean ± standard deviation (*n* = 3) of test emulsions prepared with different emulgators, different letters in columns designate statistically significant differences (*p* < 0.05).

## Data Availability

The data presented in this study are available in this article.

## Supplementary Materials

The following are available online at https://www.mdpi.com/article/10.3390/pharmaceutics16050578/s1, Figure S1: Droplet size distribution of 5% (*w*/*w*) control emulsions stabilized by: (A) P407 and mixtures BS20/P407 and (B) P407 and mixtures T60/P407, Figure S2: Droplet size distribution of 5% (*w*/*w*) control and corresponding test emulsions stabilized with: (A) P407, (B) BS20/P407 (9:1) and (C) T60/P407 (1:9), Figure S3: Droplet size distributions of 5% (*w*/*w*) emulsions measured on the 1st and 14th days after production: (A) control emulsion stabilized with P407, (B) test emulsion stabilized with BS20/P407 (9:1) (C) test emulsion stabilized with T60/P407 (1:9); Figure S4: Creaming index of control emulsions stabilized with different concentrations of P407 (monitored for the first 24 h); Figure S5: Creaming index of 5% (*w*/*w*) control emulsions stabilized with the following emulsifiers: (A) P407 and mixtures of P407 with Brij S20, (B) P407 and mixtures of P407 with Tween 60 (monitored for 7 days).
