# Supplementary material for: Characterization of Oil-in-Water Emulsions Prepared with Triblock Copolymer Poloxamer 407 and Low-Molecular-Mass Surfactant Mixtures as Carriers of Grape Pomace Waste Polyphenols
Source: Pharmaceutics. 2024 Apr 24;16(5):578. doi: 10.3390/pharmaceutics16050578 (PMC11124189; doi:10.3390/pharmaceutics16050578)
Supplement: Supplementary file 1 [file pharmaceutics-16-00578-s001.zip › pharmaceutics-2957233-supplementary.pdf]

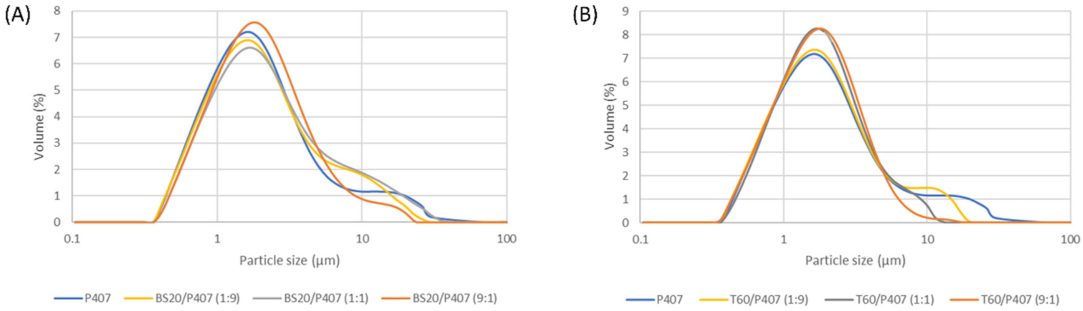

Figure S1. Droplet size distribution of 5% (w/w) control emulsions stabilized by: (A) P407 and mixtures BS20/P407 and (B) P407 and mixtures T60/P407.

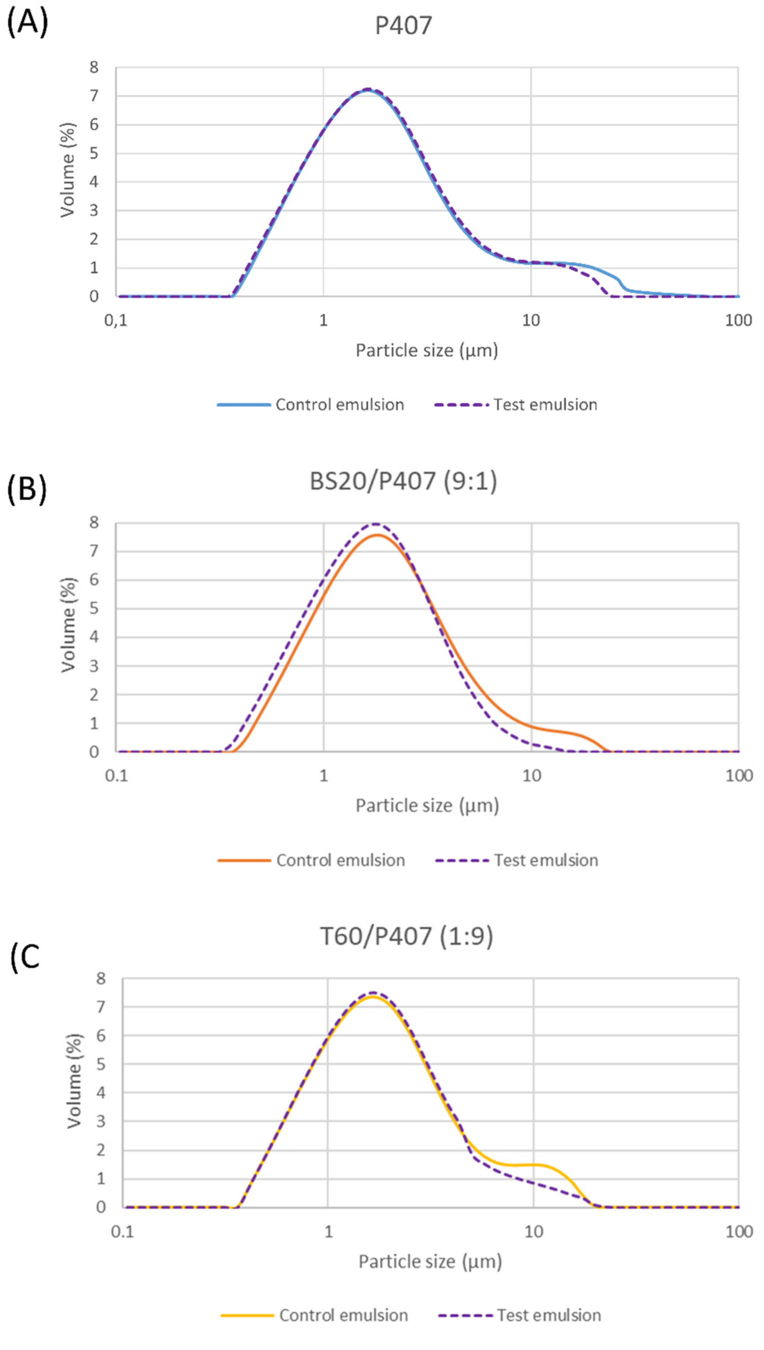

Figure S2. Droplet size distribution of 5% (w/w) control and corresponding test emulsions stabilized with: (A) P407, (B) BS20/P407 (9:1) and (C) T60/P407 (1:9).

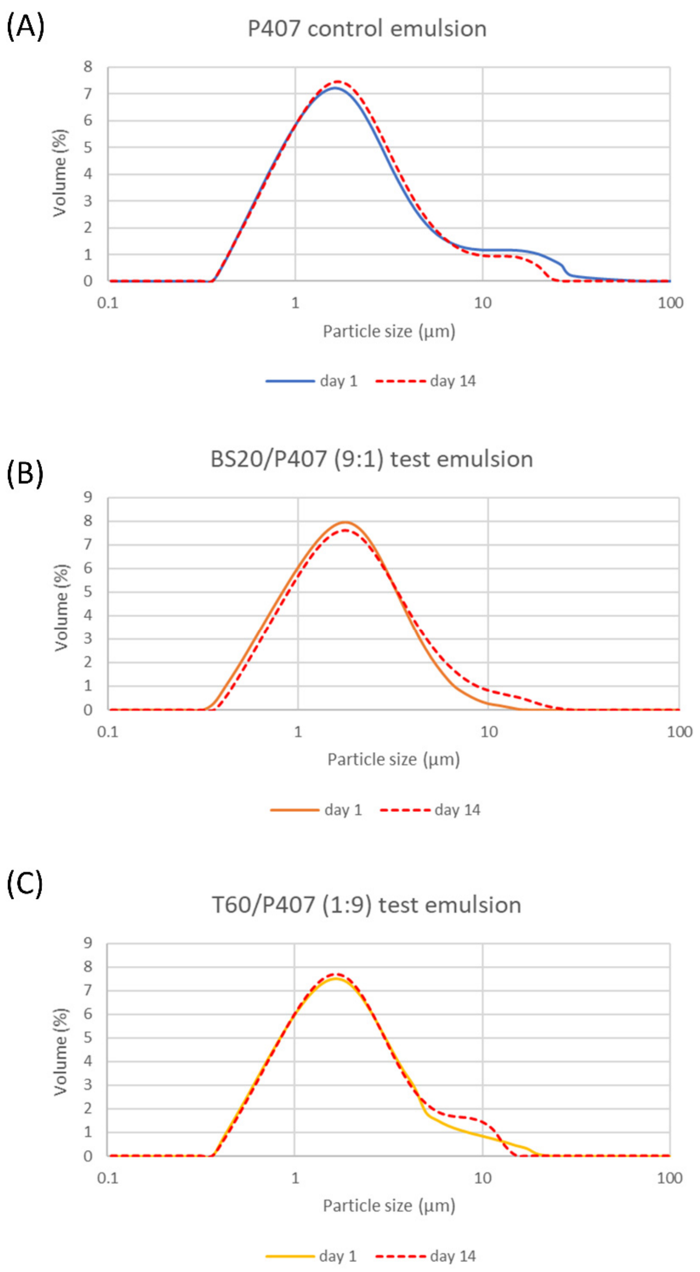

Figure S3. Droplet size distributions of 5% (w/w) emulsions measured on the 1st and 14th days after production: (A) control emulsion stabilized with P407, (B) test emulsion stabilized with BS20/P407 (9:1) (C) test emulsion stabilized with T60/P407 (1:9).

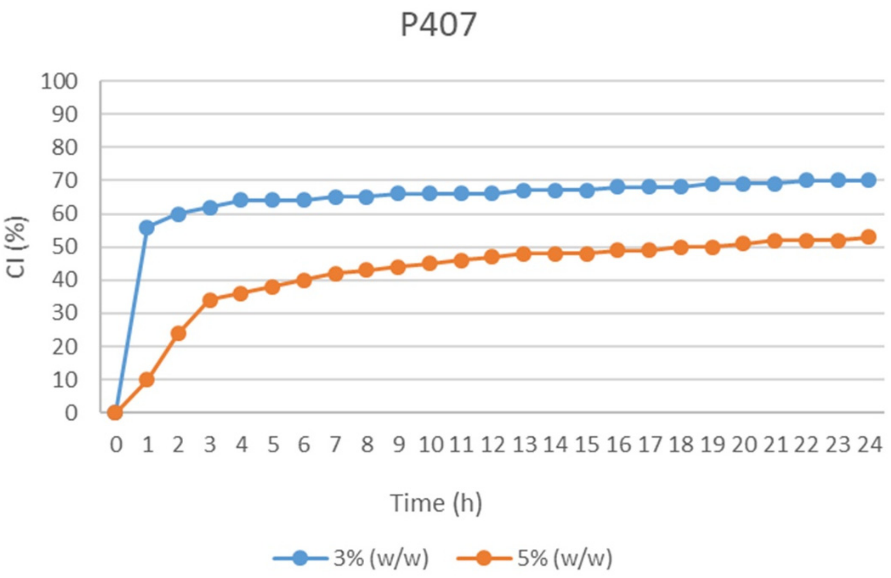

Figure S4. Creaming index of control emulsions stabilized with different concentrations of P407 (monitored for the first 24 h).

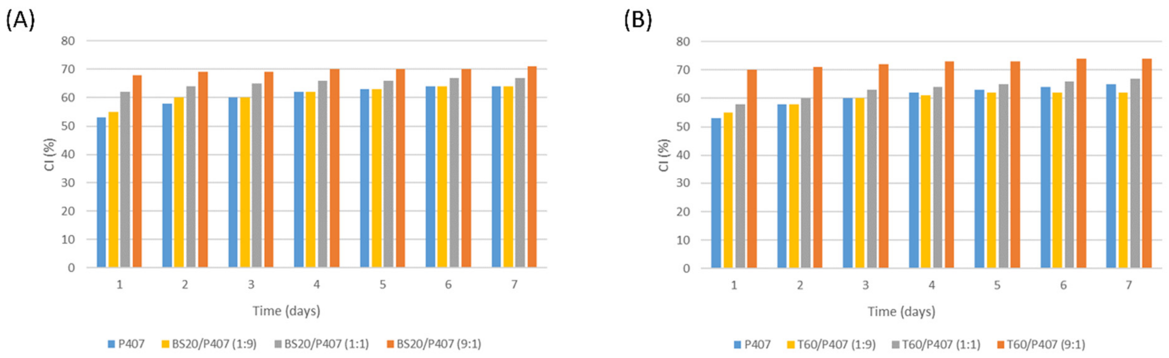

Figure S5. Creaming index of 5% (w/w) control emulsions stabilized with the following emulsifiers: (A) P407 and mixtures of P407 with Brij S20, (B) P407 and mixtures of P407 with Tween 60 (monitored for 7 days).
